# Scaling of ionic conductance in a fluctuating single-layer nanoporous membrane

**DOI:** 10.1038/s41598-023-46962-8

**Published:** 2023-11-13

**Authors:** Yechan Noh, N. R. Aluru

**Affiliations:** 1 Department of Mechanical Science and Engineering, University of Illinois at Urbana-Champaign, Urbana, IL 61801 USA; 2 Walker Department of Mechanical Engineering, Oden Institute for Computational Engineering and Sciences, University of Texas at Austin, Austin, 78712 USA

**Keywords:** Nanopores, Two-dimensional materials

## Abstract

Single-layer membranes have emerged as promising candidates for applications requiring high transport rates due to their low resistance to molecular transport. Owing to their atomically thin structure, these membranes experience significant microscopic fluctuations, emphasizing the need to explore their impact on ion transport processes. In this study, we investigate the effects of membrane fluctuations on the elementary scaling behavior of ion conductance $G$ as a function of ion concentration $c_{0}$, represented as $G = \beta c_{0}^{\alpha }$, using molecular dynamics simulations. Our findings reveal that membrane fluctuations not only alter the conductance coefficient $\beta$ but also the power-law exponent $\alpha$. We identify two distinct frequency regimes of membrane fluctuations, GHz-scale and THz-scale fluctuations, and examine their roles in conductance scaling. Furthermore, we demonstrate that the alteration of conductance scaling arises from the non-linearity between ion conductance and membrane shape. This work provides a fundamental understanding of ion transport in fluctuating membranes.

## Introduction

The atomic thickness of 2D membranes offers minimal resistance to molecular transport, making them favorable for applications that demand a high transport rate. Hence, 2D membranes have been actively studied for a wide range of membrane applications such as water desalination^1–8^, electricity generation from salinity gradient^9–12^, and separation of heterogeneous fluids^13–15^. In particular, the transport of charged particles, including ions, is of significant interest due to their relevance in energy applications^9–11^ and numerous biological processes^16–21^. Unlike the obvious linear scaling between conductance and concentration of ions in a macroscopic pore, $G\propto {c}_{0}$, the transport of ions in a nanoscale pore involves unique scaling behavior at low concentration^22–26^, $G\propto {c}_{0}^{\alpha }$, where $G$ is the ion conductance, ${c}_{0}$ is the ion concentration, $\alpha$ is the power-law exponent, and $\beta$ is the coefficient. Understanding the scaling behavior is an elementary step towards understanding the nanoscale ion transport phenomena. However, the current understanding of conductance scaling is limited to rigid nanopores, where the structural flexibility of nanopore is neglected. Given their thinness, atomically-thin membranes exhibit significant structural fluctuations, which are known to considerably affect both water permeation^1,27,28^ and ion transport^29,30^. Thus, a fundamental understanding of how these fluctuations influence the scaling behavior of ion conductance is important, especially considering the growing interest in 2D membrane applications.

Ion conductance in a bulk solution exhibits linear scaling between conductance and ion concentration, represented by $G\propto {c}_{0}$. However, ion conduction near a solid surface deviates from this linear scaling. For narrow pores or channels, various scaling behaviors have been reported, ^23,31–33^, especially where the surface contribution to ion conductance is significant. In 1993, Lev et al. reported deviations in the conductance scaling at low ion concentrations, attributing this phenomenon to various surface effects such as ion concentration differences due to surface charge, direct ion adsorption to the surface, and ordered structures of the ionic solution near the surface. Stein et al.^23^ in 2004 reported a fully saturated ion conductance at low ion concentration (i.e., zero power-law exponent, $\alpha =0$) in silica channels ranging from 1 µm to 70 nm. They demonstrated a direct relationship between the surface charge density of the channel and the saturated conductance, suggesting that saturation occurs due to the transport of counter-ions near the channel surface. Electrokinetic theories^32,33^ have been proposed and utilized to estimate the surface charge density of nanochannels and nanopores^9,26,34–36^. However, these models do not universally account for the observed behaviors in all systems. Notably, conductance does not always fully saturate.

Previous studies have identified a few attributes that influence the various scaling behavior of ion conductance. One attribute is the charge regulation, while another is the leakage of surface potential out of the pore. Charge regulation refers to the phenomenon at the solid–fluid interface where the surface charge density avries as a function of ion concentration and effects the scaling behavior of the surface conduction. Several charge regulation models have been proposed to explain the various power-law exponents measured^24,25,37,38^. Another phenomenon that affects the conductance scaling is the pore potential leakage. When the potential generated by the surface charge leaks, it leads to a distribution of counterions both inside and outside the pore. Counterions positioned outside the pore do not directly contribute to the surface conduction. This leads to diverse scaling behaviors, depending upon the extent of the potential leakage. Specifically, ion conductance scaling studies conducted using Molecular Dynamics (MD) simulations have shown that the power-law exponent can vary from zero to one depending on the degree of electric potential leakage^22^. The simulations suggest that the power-law exponent approaches zero for nanotubes/pores with low radius/length ratio where the potential leakage is negligible. On the other hand, for nanotubes/pores with high radius/length ratio, such as 2D nanopore membranes with diameter larger than 2 nm, the pore potential leakage become significant, resulting in various power-law exponents depending on the degree of the potential leakage. It is noted that although many nanopores conform to this qualitative trend, including some biological nanopores—which are considered highly flexible compared to synthetic nanopores—the power-law exponent in biological nanopores^25^ is approximately 0.05–0.2 smaller than that in other nanopores with similar aspect ratios^22^. Some experimental data, such as those from CNT^24^ and nanofilters made of polyethylene terephthalate^31^ cannot be solely explained by this phenomenon, implying that the conductance scaling is a complicated phenomenon associated with multiple factors, such as charge regulation and nanopore flexibility.

In this work, we investigate the effect of membrane fluctuations on the scaling behavior of ion conductance using MD simulations. We demonstrate that nanopore membrane fluctuations can influence both the power-law exponent and coefficient of ion conductance. We introduce two distinct frequency regimes (GHz-scale and THz-scale) of membrane fluctuations and examine the contribution of each vibrational mode to the ion conductance.

## Results and discussions

**Figure 1:**
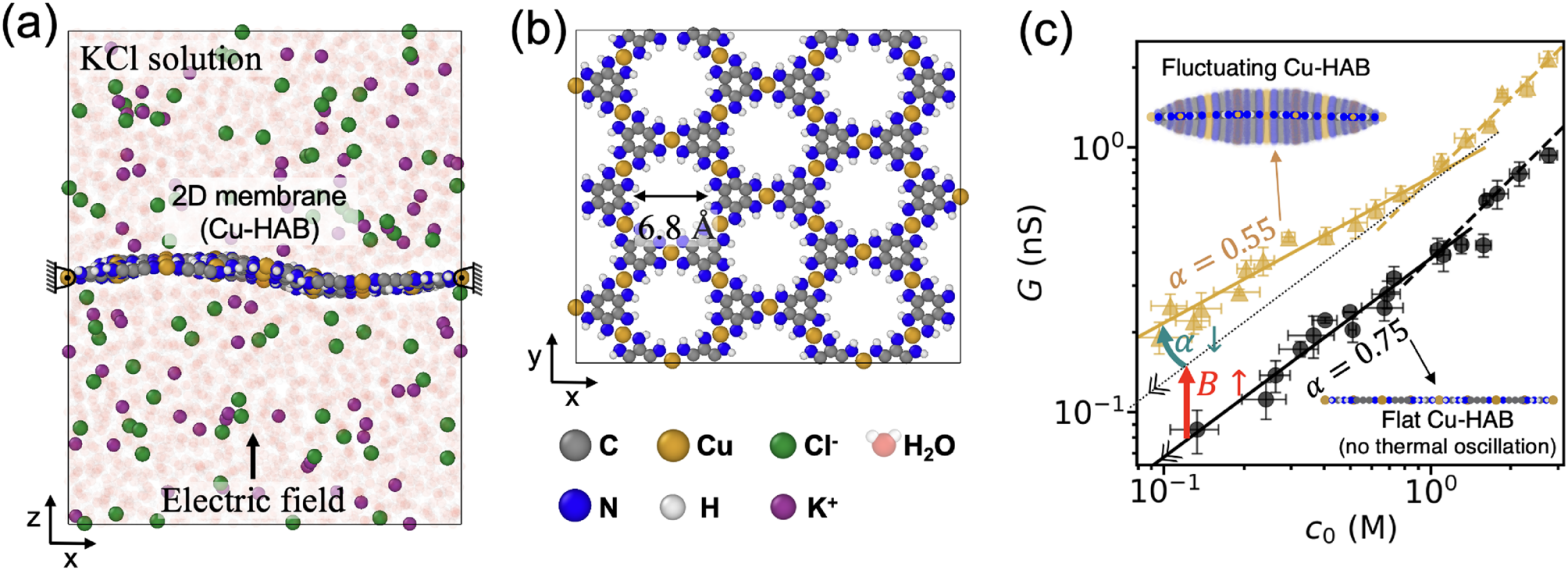
MD simulation of ionic conduction in membranes with and without fluctuations. (**a**) Illustration of the simulation setup. (**b**) Top view of Cu-HAB membrane. (**c**) Ionic conductance as a function of reservoir ion concentration for a fluctuating and non-fluctuating Cu-HAB membrane. In the sub-figure, the transparent color of the membrane represents the fluctuations and the solid color represent the mean displacement. The figure is plotted in logarithmic scale. The error bars represent the standard error of the conductance values obtained from each individual 1 ns dataset.

To investigate the impact of membrane fluctuations on the scaling of ion conductance, we conducted MD simulations of ion transport through a single layer of nanoporous membrane (Cu-HAB^1,39,40^) as shown in Fig. 1a, b. The nanoporous membrane is immersed in a KCl solution. The edges of the membrane are fixed, and the rest of the membrane experiences thermal fluctuations during the simulation. For comparison, we also considered a rigid counterpart where the membrane fluctuations are suppressed, maintaining a flat configuration throughout the simulation. To generate ion transport across the membrane, an electric field is applied perpendicular to the membrane. The ion conductance is obtained for various concentrations of KCl to investigate the scaling behavior between ion conductance and ion concentration. The details on MD simulations can be found in the “Methods” section. As stated in the introduction section, the relationship between ion conductance and concentration in nanopore membranes can be characterized by a power-law equation of the form, $G=\beta {c}_{0}^{\alpha }$. Our simulations show that the membrane fluctuations alter both the coefficient of conductance $\beta$ and the power-law exponent $\alpha$, as shown in Fig. 1c. Specifically, the membrane fluctuation increases the coefficient $\beta$ by approximately 100% and reduces the power-law exponent from 0.75 to 0.55. We note that the conductance scaling is reduced only in the low concentration regime where the surface conductance is dominant. In the high concentration regime, where the bulk transport mechanism is dominant, we observed a linear scaling (i.e., $\alpha =1$) for membranes with and without fluctuations. However, the power-law exponent is reduced in the low concentration regime where the surface transport mechanism is dominant. It is noted that this modification of power-law exponent results in a higher discrepancy in conductance with and without membrane fluctuations as the concentration decreases: 102% at 1.0 M, 221% at 0.1 M, and 409% at 0.01 M. These results suggest that the membrane fluctuations can significantly impact ionic conductance for low ion concentration environments.

**Figure 2:**
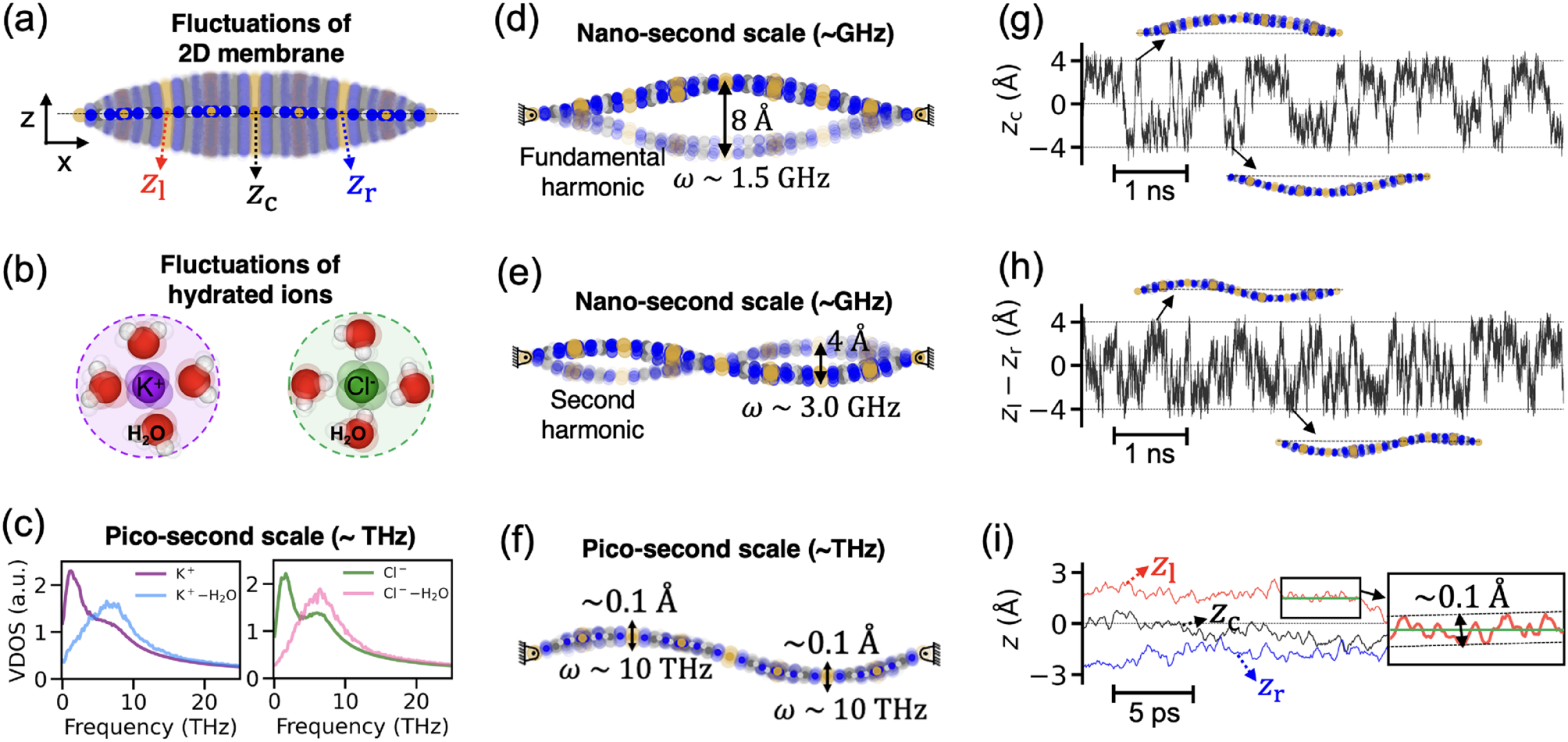
Fluctuations of membrane and hydrated ions. (**a**) Comprehensive fluctuations of Cu-HAB membrane with fixed ends. The solid color represents the mean displacement of the membrane, and the transparent color represents the fluctuations of membrane. ${z}_{\mathrm{l}}$, ${z}_{\mathrm{c}}$, and ${z}_{\mathrm{r}}$ represent the out-of-plane displacement at one-quarter, center, and three-quarter of the membrane length, respectively (measured from the left end). (**b**) Illustration of the fluctuation of ions and hydration water. (**c**) Vibrational density of states of ions and ion-hydration water stretching mode. (**d**) A fundamental harmonic mode and (**e**) a second harmonic mode of GHz-scale fluctuations. (**f**) THz-scale oscillations of membrane. (**g**) Displacement of the center of the membrane ${z}_{\mathrm{c}}$ over a period of few nanoseconds. (**h**) Displacement of the one-quarter point ${z}_{\mathrm{l}}$ and three-quarter point ${z}_{\mathrm{r}}$. (**i**) Displacement of the membrane ${z}_{\mathrm{l}}$, ${z}_{\mathrm{c}}$, and ${z}_{\mathrm{r}}$ over a period of few tens of picoseconds.

To better understand the impact of membrane fluctuations on the scaling of ion conductance, we analyzed the fluctuations of the membrane and the hydrated ions. Figure 2a shows a visualization of membrane fluctuations obtained by superimposing multiple snapshots of membranes. The solid color represents the mean displacement of the membrane and the transparent color represents the fluctuations. The mean displacement of the membrane is approximately zero and the amplitude of fluctuation varies with the x-coordinate of the membrane. The maximum fluctuation in the amplitude is observed to be 4 Å, which is comparable to the diameter of the nanopore (6.8 Å). This implies that membrane fluctuations become important when the critical length scale involving transport processes is of the order of a few nanometers or below. In addition to the membrane fluctuations, we also examined the fluctuations of hydrated ions and their vibrational frequencies. Figure 2b shows the fluctuations of hydrated ions and Fig. 2c its vibrational density of states (VDOS). The fluctuations of ions surrounded by water molecules involves THz scale fluctuations^41^. Specifically, the vibrations of ${\mathrm{K}}^{+}$ and ${\mathrm{Cl}}^{-}$ exhibits their peaks at $1.2 \mathrm{THz}$ and $1.6 \mathrm{THz}$, respectively. Also, ${\mathrm{K}}^{+}-{\mathrm{H}}_{2}\mathrm{O}$ and ${\mathrm{Cl}}^{-}-{\mathrm{H}}_{2}\mathrm{O}$ stretching mode in the first hydration shell exhibit their peaks at $6.2 \mathrm{THz}$ and $6.4 \mathrm{THz}$, respectively.

To understand the effect of an individual vibrational mode of the membrane on the ion conductance, we first decomposed the membrane fluctuations into several individual modes. We investigated the trajectories of membranes at the picosecond to nanosecond scale and identified two separate frequency regimes of membrane fluctuations: gigahertz (GHz)-scale and terahertz (THz)-scale fluctuations. The GHz-scale fluctuations involve the wiggling motion of the membrane as a result of the collective motion of membrane atoms. This mode of fluctuation is much slower than the fluctuations of ions and its hydration water. On the other hand, THz-scale frequencies are due to the trembling oscillations of membrane atoms, and this scale of frequency is comparable to the frequency of the fluctuations of the hydrated ions. We found that GHz-scale fluctuations mainly involve the first and second modes of standing waves with fixed edges (i.e., the fundamental harmonic and second harmonic modes), as shown in Fig. 2d, e. To estimate the amplitude and the frequency of the fundamental harmonic mode, we analyzed the z-coordinate of the center of the membrane ${z}_{\mathrm{c}}$. Figure 2g shows the fluctuations of ${z}_{\mathrm{c}}$ over time and this indicates that the amplitude of the fundamental harmonic mode is approximately 4 Å. The fundamental harmonic frequency is approximately 1.5 GHz based on the average frequency of ${z}_{\mathrm{c}}$ alterations between 4 and − 4 Å. Similarly, we determined the amplitude and frequency of the second harmonic mode by analyzing $z_{{\text{l}}} - z_{{\text{r}}}$ (see Fig. 2h). The estimated amplitude of the second harmonic mode is around 2 Å, with a frequency of approximately 3.0 GHz. Compared to the first harmonic mode, the amplitude of the second harmonic is halved, and its frequency is doubled. Note that this range of GHz frequency is three orders of magnitude slower than the frequency of the hydrated ions, indicating that the wiggling motion of membrane has a negligible vibrational coupling with the oscillation of hydrated ion. While there are also THz scale fluctuations of the membrane as illustrated in Fig. 2f, this scale of fluctuation is due to the thermal oscillation of membrane atoms. The typical scale of the amplitude and the frequency is ~ 0.1 Å and ~ 10 THz (see Fig. 2i), which is comparable to the fluctuations of ions and its hydration water. Therefore, the thermal oscillation of membrane atoms can create a meaningful vibrational coupling with nearby ions.

**Figure 3:**
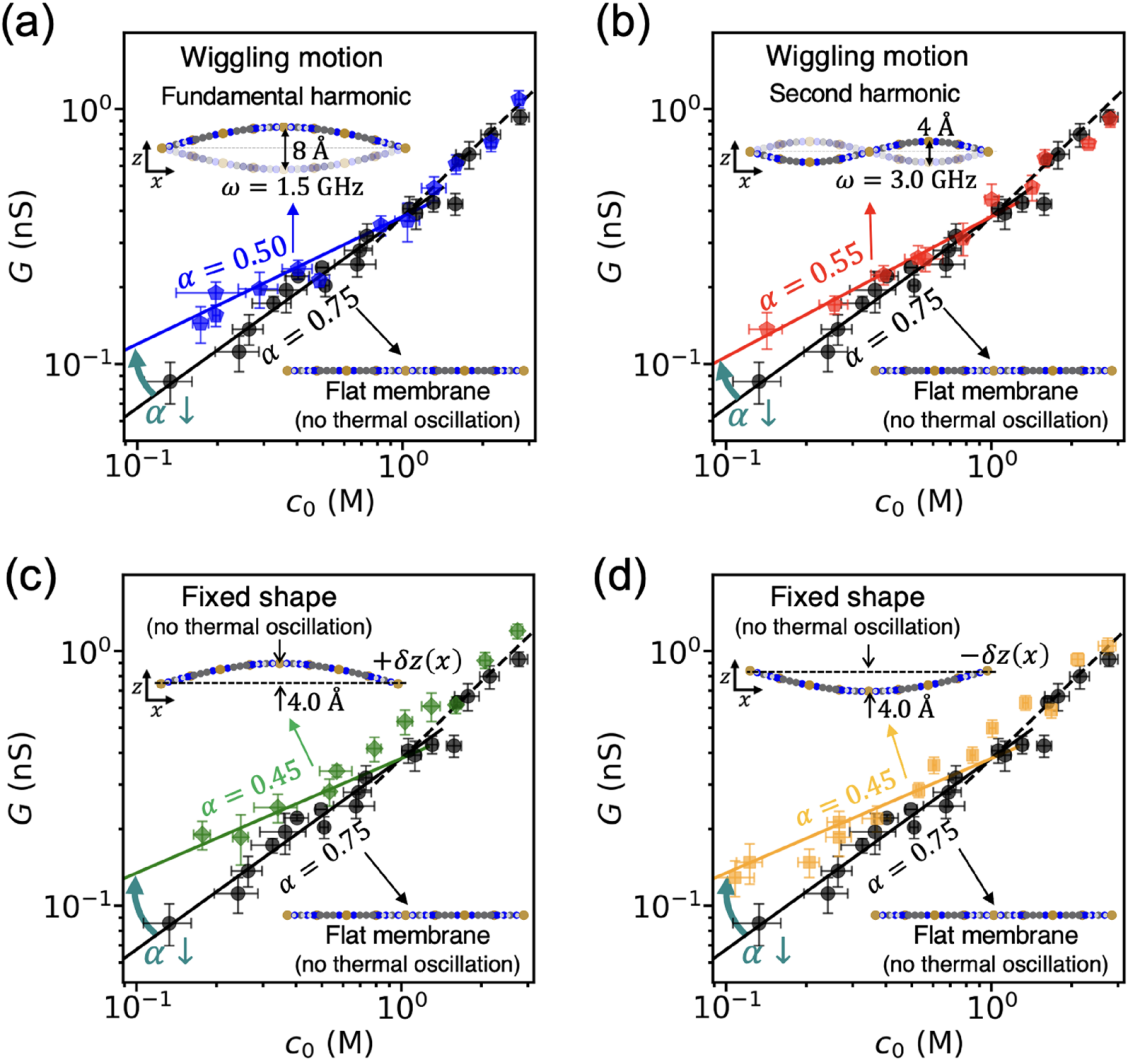
Effect of GHz membrane fluctuation on the conductance scaling and observation of non-linearity arising from the shape of membrane. Tuning of conductance scaling due to (**a**) the fundamental harmonic mode and (**b**) the second harmonic mode of wiggling motion of membrane. Ion conductance and ion concentration are plotted on a logarithmic scale. (**c**) and (**d**) A nonlinear relationship between the membrane shape and conductance. The error bars represent the standard error of the conductance values obtained from each individual 1 ns dataset.

Next, we examined how the individual modes of membrane fluctuations influence the ion conductance and its scaling. Toward this, we designed conceptual studies for membranes with specific modes of fluctuation. Specifically, we first explored the effect of GHz-scale fluctuations on the scaling of ion conductance. The wiggling motion of membrane is modeled with the standing wave equation for a string with two fixed edges, which is given by $A\mathrm{sin}\left\{n\pi \left(\frac{x}{L}+\frac{1}{2}\right)\right\}\mathrm{sin}\left(2\pi \omega t\right)$, where $A$ is the amplitude, $n$ is the mode number, $L$ is the length of the membrane along the x-coordinate $\left(-\frac{L}{2}\le x\le \frac{L}{2}\right)$, $\omega$ is the frequency, and $t$ is the time. Figure 3a, b show the conductance-concentration curves under the GHz-scale wiggling motions controlled by the standing wave equation, with amplitudes of 4 Å (Fig. 3a) and 2 Å (Fig. 3b) and L = 4.62 nm. Note that in these controlled wiggling motions, the trembling oscillations of atoms are suppressed, allowing us to exclusively study the effects of the membrane's wiggling motions on ion transport. Notably, the power-law exponent is reduced under the controlled wiggling motion for both the first and second harmonic modes, compared to its rigid counterpart where the thermal fluctuations are suppressed and the membrane remains flat during the simulation. This demonstrates that the wiggling motion of the membrane alters the scaling of ionic conductance. The wiggling motion of the membrane is in the GHz frequency range and that is significantly slower—by three orders of magnitude—compared to the frequencies associated with the fluctuations of hydrated ions which are in the THz-scale. Therefore, the reduced power-law exponent $\alpha$ due to the GHz-scale wiggling motion does not appear to be a result of vibrational coupling between the membrane and nearby electrolyte.

Our next step will be to examine the physical mechanism underlying the reduced power-law exponent $\alpha$, created by GHz-scale wiggling motion of the membrane. In these simulations, charge regulation is excluded (surface charge is fixed as a constant). Thus, we focused on the leakage of a surface potential, which is closely associated with the geometry of the pore/channel. In the case of long channel that has an enough space to accommodate counter ions attracted to the channel surface, the effect of surface potential leakage is trivial resulting in the saturation of ion conductance at low concentration (i.e., $\alpha \approx 0$). In contrast, a thin pore, such as a 2D membrane, lacks adequate space to accommodate the counter ions. As a result, the surface potential leaks out of the pore, resulting in various power-law exponents between zero and one, depending on the degree of leakage. Given the importance of nanopore geometry in ion conductance scaling, we investigated whether changes in membrane shape, resulting from GHz-scale wiggling motions, could impact the power-law exponent. Notably, the mean displacement of the GHz-scale fluctuation is zero. Therefore, the potential non-linearity between ion conductance scaling and membrane shape needs to be examined.

**Figure 4:**
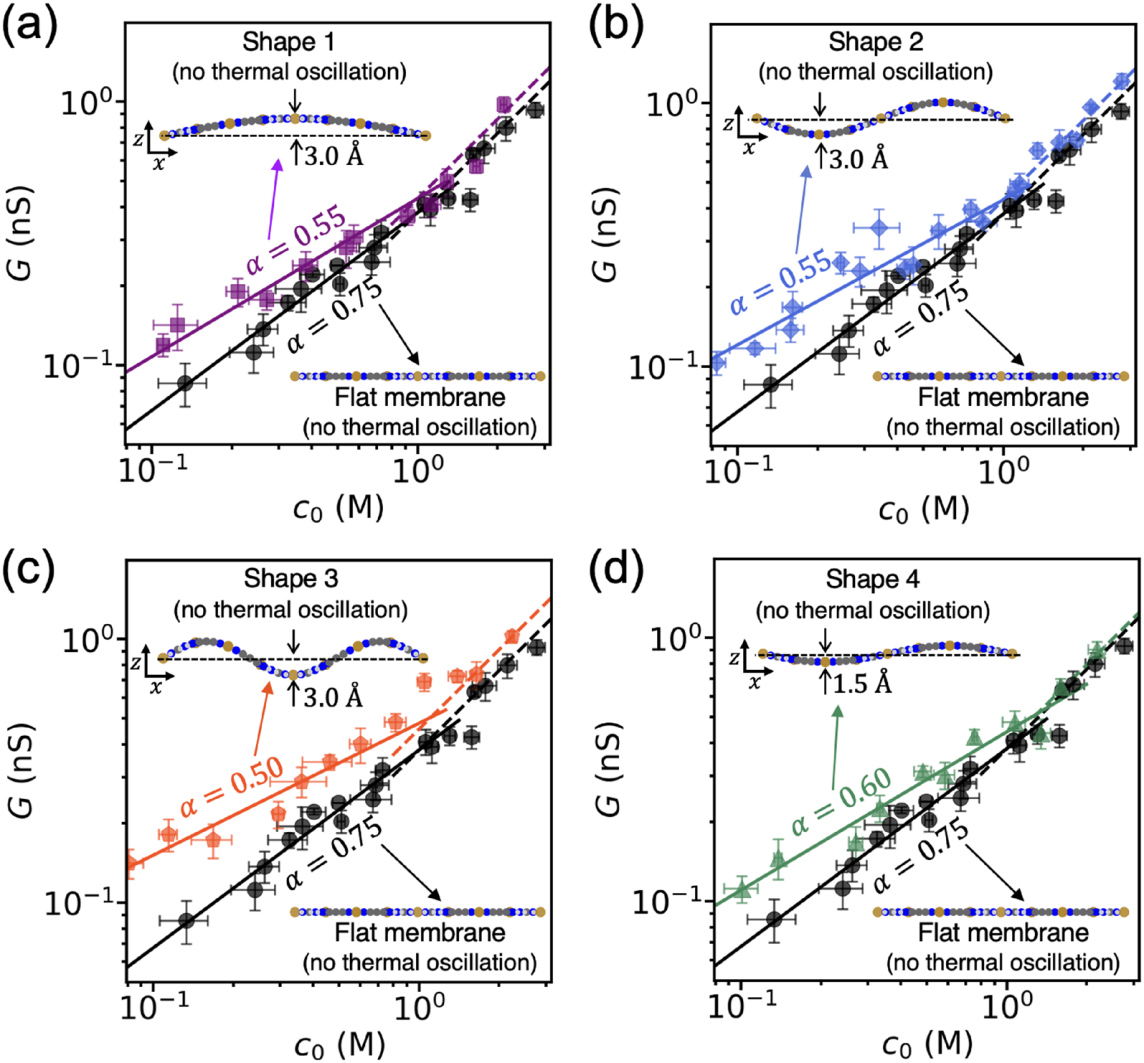
Conductance scaling for various shapes of membranes. (**a**) A fundamental harmonic shape with a displacement of 3.0 Å. (**b**) a second harmonic shape with a displacement of 3.0 Å. (**c**) a third harmonic shape with a displacement of 3.0 Å. (**d**) a second harmonic shape with a displacement of 1.5 Å. All the membrane shapes are fixed and do not change during the simulation. The error bars represent the standard error of the conductance values obtained from each individual 1 ns dataset.

To test the linearity, we considered two membranes with opposite shapes, $\delta z\left(x\right)$ and $-\delta z\left(x\right)$ (see the subfigure of Fig. 3c, d), so that the summation of the displacements is zero (flat membrane). To exclusively study the effect of the membrane shape, we fixed the membrane shape during the simulation and suppressed thermal oscillations of the membrane atoms. This case study differs from the previous study of a wiggling membrane where the membrane shape changed over time. If the conductance is linear with the displacement of the membrane, the linear relation, $G\left(z=\delta z\left(x\right)\right)+G\left(z=-\delta z\left(x\right)\right)=G\left(z=0\right)$, must hold. The simulation results for these two opposite shapes of membrane are shown in Fig. 3c, d. Both membranes yield a reduced power-law exponent compared to the flat membrane. Note that the linearity equation does not hold, which demonstrates non-linearity in the conductance scaling with respect to the membrane shape. This non-linearity explains that zero-mean wiggling motions of membrane can reduce the power-law exponent. To further understand how various shapes of membrane affect conductance scaling, we examined membranes with several different shapes. The results in Fig. 4 consistently show that the membrane shape can reduce the power-law exponent and slightly increase the coefficient of ion conductance $\beta$. Our data shows that the amount of displacement is one of the factors for this effect (see Fig. 4b–d). We further analyzed the ion concentration at the pore, ${c}_{\mathrm{p}}$, and compared it to the concentration at the reservoir. Supplementary Fig. 1 indicates that the deformation of the membrane shape influences the scaling of ion concentration at the pore. This suggests that deformed membranes exhibit less electrical potential leakage compared to flat membranes^22^. These results suggest that ion conductance can be tuned by modifying membrane shape, which has potential applications in a variety of nanofluidic devices.

**Figure 5:**
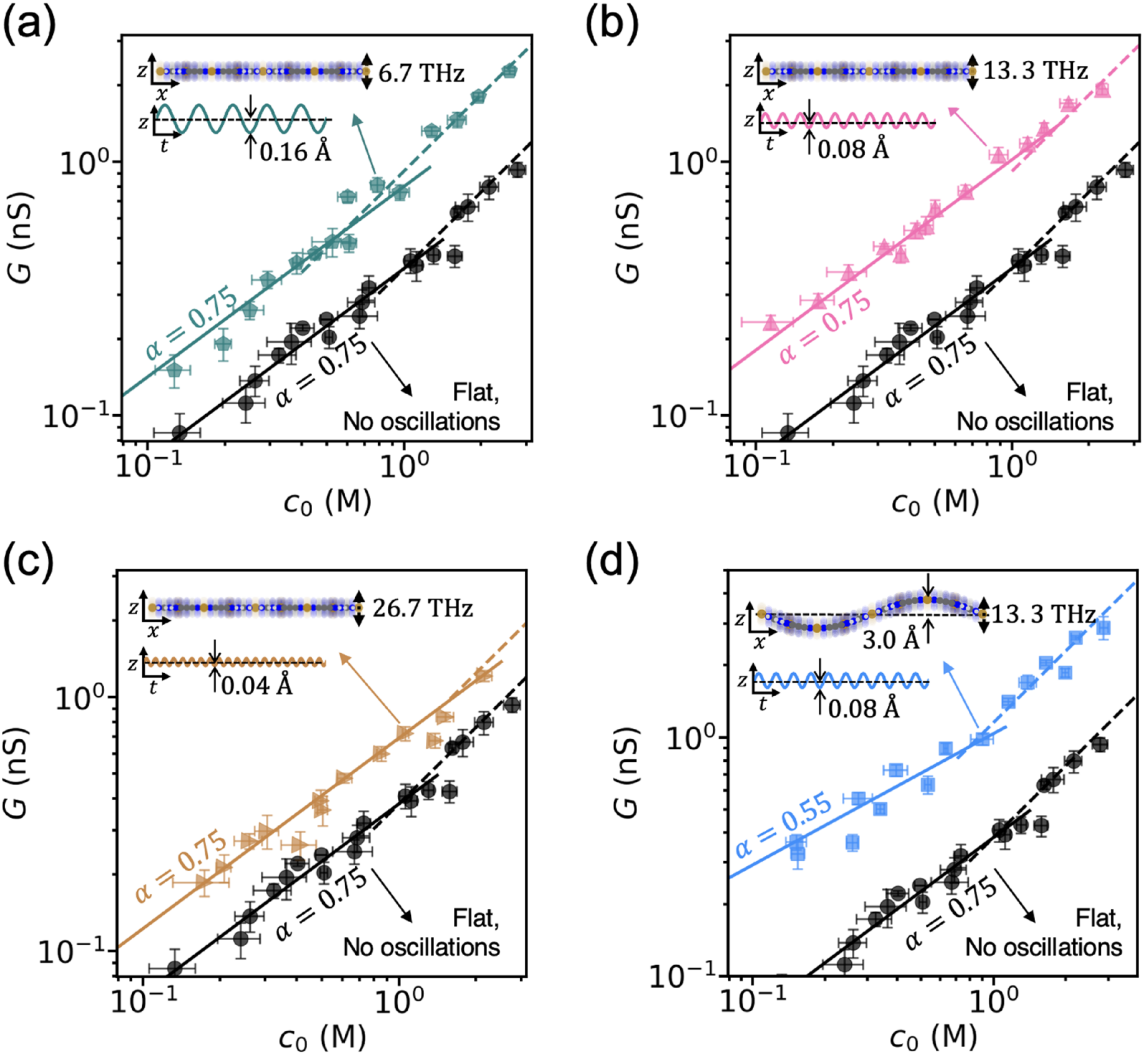
Effect of THz-scale oscillations of membrane on the conductance scaling. Ion conductance scaling is shown for a flat membrane with frequencies of (**a**) 6.7 THz, (**b**) 13.3 THz, and (**c**) 26.7 THz. (**d**) Ion conductance scaling is shown for a wrinkled membrane oscillating at a frequency of 13.3 THz. The shape of the membrane is fixed during the simulation and the entire membrane is oscillating with a certain frequency and amplitude depicted in each figure. All figures are plotted on a logarithmic scale. The error bars represent the standard error of the conductance values obtained from each individual 1 ns dataset.

Finally, we studied the impact of THz-scale oscillations of membrane atoms on ion conductance. For this, we applied a sinusoidal function with a particular frequency and amplitude to oscillate the entire membrane. To investigate the effect of THz-scale oscillations of membrane, the shape of the membrane is fixed during the simulation. Figure 5 illustrates the effects of THz-scale membrane oscillations on the conductance-concentration curve. The simulation results indicate that the THz-scale oscillations of the membrane do not alter the power-law exponent $\alpha$ (see Fig. 5a–c). Figure 5d shows a case with the same shape used in Fig. 4b, which yields the same power-law exponent $\alpha =0.55$. This further supports the result that THz-scale oscillations of the membrane do not alter the power-law exponent, but they can considerably increase the coefficient of ionic conductance $\beta$, in contrast with the GHz-scale wiggling motions of the membrane. The increase of the coefficient can be understood as a result of the vibrational coupling between the membrane and nearby hydrated ions as our earlier studies suggest^30,42^.

## Conclusions

Scaling of ionic conductance in fluctuating 2D membranes is an important area of research due to its potential applications in membrane-based technologies. In this study, we investigated the effect of membrane fluctuations on ionic conductance using molecular dynamics simulations. Our findings indicate that fluctuations in 2D membranes have an impact on the scaling behavior of ion conductance. Specifically, we found that membranes experiencing fluctuations have a lower power-law exponent and a higher coefficient of conductance compared to those without fluctuations. To better understand how membrane fluctuations affect conductance, we first analyzed the membrane fluctuations and observed two distinct ranges of membrane fluctuations. One is GHz-scale frequency mode which is due to the wiggling motions of membrane and the second is THz-scale frequency mode that involves the thermal vibration of atoms. We then conducted conceptual studies with controlled membrane fluctuations with a specific vibrational mode. The results show that the GHz-scale wiggling motion of a membrane reduces the power-law exponent. Our simulations showed a non-linear relationship between the conductance scaling and the membrane shape, suggesting that the fluctuation of a membrane can alter the conductance scaling even though the mean displacement is zero. On the other hand, THz-scale fluctuations do not alter the power-law exponent, but considerably increase the coefficient of the ion conductance. This study provides a fundamental understanding of how different vibrational modes of the membrane effect ion transport. Our findings suggest that modifying the shape of membranes could be a feasible method for tuning ionic conductance in membrane applications.

## Methods

We conducted molecular dynamics (MD) simulations to investigate the scaling of ion conductance in fluctuating 2D nanoporous membranes. Specifically, our simulations focused on a Cu-HAB membrane^1,39^ with dimensions of 4.62 nm by 4.00 nm, containing 12 pores in a periodic cell. The edges of the membrane were fixed, while the rest of the membrane was allowed to fluctuate during the simulations. To facilitate comparison, we also conducted ion transport simulations for a rigid membrane, where all membrane atoms were fixed. To generate an electric potential gradient, a uniform external electric field (0.08632 V/nm) perpendicular to the membrane was applied, and the system was maintained at a uniform dimension of 5.80 nm perpendicular to the membrane. We modeled the KCl solution using Coulombic and Lennard–Jones potentials, and the ReaxFF force field^43^ was used to model the membrane. The partial charges of the membrane are calculated by the charge equilibration (QEq) method^44^ and fixed them during the simulation. We used the particle–particle particle-mesh method^45^ to calculate long-range Coulombic interactions. We adopted LJ parameters from^46–48^ and calculated LJ parameters between two different atoms using the Lorentz-Berthelot combination rule, with the LJ potential cut-off distance set at 1.2 nm. The membrane-liquid interactions were modeled using LJ and Coulombic potentials, while we used the flexible simple point charge water model for water^49^. The potential parameters described by Joung et al.^50^ were used for ions. The system temperature is maintained using the Nosé–Hoover thermostat, and atomic trajectories were computed using an NVT ensemble with a time interval of 0.5 fs. We examined several cases where the membrane was subjected to a specific vibrational mode. To model GHz-scale fluctuations, we utilized the standing wave equation of the fundamental and second modes with the specific frequencies and amplitudes described in the main text. For THz-scale vibrations, the membrane was assumed to be rigid and excited sinusoidally with a particular amplitude and frequency described in the main text and Fig. 5. We measured ion current by counting the number of ions passing through the membrane, with equilibration conducted for 1 ns and data collection for 5–10 ns. Then the ion conductance is calculated as $G = I/{\Delta }V$, where $I$ is ion current and ${\Delta }V$ is cross-membrane electric potential difference. VDOS is calculated as ${\text{VDOS}}\left( \omega \right) = \mathop \smallint \limits_{ - \infty }^{\infty }\langle v\left( {t + t_{0} } \right)v\left( {t_{0} } \right)\rangle e^{ - 2\pi i\omega t} dt$ where $v$ is the time derivative of position (e.g., relative position of ion and water in the first hydration shell for water-ion stretching mode), $t_{0}$ is the reference time, $\omega$ is the frequency, and the angle brackets denote the time and ensemble average. MD simulations are performed by LAMMPS and used OVITO^51^ for atomic visualizations.

### Supplementary Information

Supplementary Information 1.

Supplementary Figure 1.

## Data Availability

All data generated or analysed during this study are included in this published article [and its supplementary information files].
