# Supplementary material for: Scaling of ionic conductance in a fluctuating single-layer nanoporous membrane
Source: Sci Rep. 2023 Nov 13;13:19813. doi: 10.1038/s41598-023-46962-8 (PMC10643653; doi:10.1038/s41598-023-46962-8)
Supplement: Supplementary file 2 — Supplementary Figure 1. [file 41598_2023_46962_MOESM2_ESM.pdf]

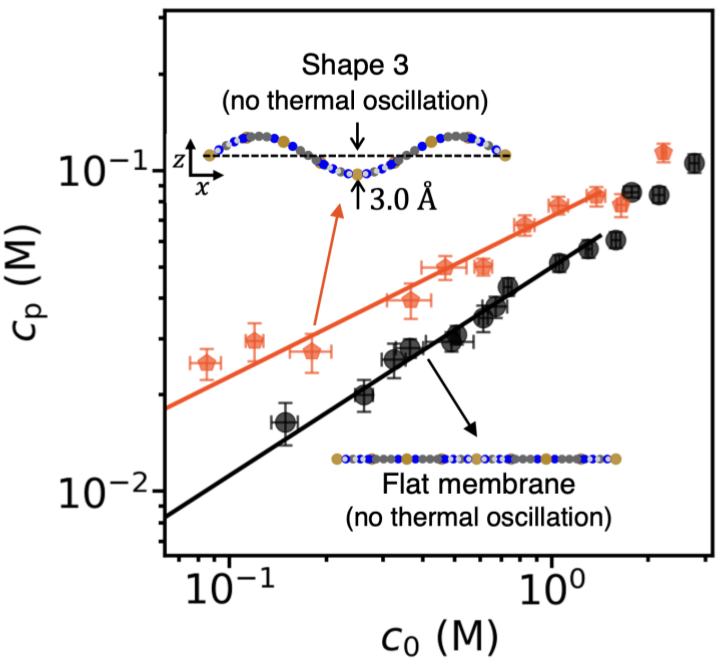


Supplementary Figure 1. Ion concentration at the pore versus ion concentration at the reservoir. The ion concentration at the pore is obtained using a sphere centered at the pore center with a radius of 4.42 Å. The reservoir concentration is calculated from the side of the simulation box with a thickness of 1.0 nm. Error bars represent the standard error in concentration obtained from an individual 1 ns dataset.
